# Basic Blue Skies Research in the UK: Are we losing out?

**DOI:** 10.1186/1747-5333-3-3

**Published:** 2008-02-29

**Authors:** Belinda Linden

**Affiliations:** 1 12 Balcaskie Road, Eltham, London SE9 1HQ, UK

## Abstract

**Background:**

The term blue skies research implies a freedom to carry out flexible, curiosity-driven research that leads to outcomes not envisaged at the outset. This research often challenges accepted thinking and introduces new fields of study. Science policy in the UK has given growing support for short-term goal-oriented scientific research projects, with pressure being applied on researchers to demonstrate the future application of their work. These policies carry the risk of restricting freedom, curbing research direction, and stifling rather than stimulating the creativity needed for scientific discovery.

**Methods:**

This study tracks the tortuous routes that led to three major discoveries in cardiology. It then investigates the constraints in current research, and opportunities that may be lost with existing funding processes, by interviewing selected scientists and fund providers for their views on curiosity-driven research and the freedom needed to allow science to flourish. The transcripts were analysed using a grounded theory approach to gather recurrent themes from the interviews.

**Results:**

The results from these interviews suggest that scientists often cannot predict the future applications of research. Constraints such as lack of scientific freedom, and a narrow focus on relevance and accountability were believed to stifle the discovery process. Although it was acknowledged that some research projects do need a clear and measurable framework, the interviewees saw a need for inquisitive, blue skies research to be managed in a different way. They provided examples of situations where money allocated to 'safe' funding was used for more innovative research.

**Conclusion:**

This sample of key UK scientists and grant providers acknowledge the importance of basic blue skies research. Yet the current evaluation process often requires that scientists predict their likely findings and estimate short-term impact, which does not permit freedom of research direction. There is a vital need for prominent scientists and for universities to help the media, the public, and policy makers to understand the importance of innovative thought along with the need for scientists to have the freedom to challenge accepted thinking. Encouraging an avenue for blue skies research could have immense influence over future scientific discoveries.

## Introduction

"Scientific investigations and experimental ideas may have their birth in almost involuntary chance observations which present themselves either spontaneously or in an experiment made with a different purpose."

Claude Bernard 1927 [1].

Basic 'Blue skies' research has been described as pure science, exploratory, innovative, curiosity-driven and fundamental, in contrast with goal-driven research [2]. The terms 'basic research' and 'blue skies research' have often been used interchangeably, particularly when discussing funding. This is discussed in the work of Calvert, who explores the various ways that these terms are understood by scientists and policy makers [3].

The Frascati definition (1963) for basic research is:

*"Experimental or theoretical work undertaken primarily to acquire new knowledge of phenomena and observable facts without any particular application or use in view. It is usually undertaken by scientists who may set their own agenda and to a large extent organise their own work *[4]*."*

This definition is similar to three interpretations of blue skies research:

*"Excellent curiosity-driven science, free from boundaries *[5]*."*

*"Innovative and creative research that might lead to outcomes unimagined at the outset *[6]*."*

*"Addresses fundamental, curiosity driven science *[7]*."*

Therefore, its main goal is to advance knowledge and understanding. It is often exploratory – driven by a researchers' interest or curiosity. It may not have a practical goal in mind and yet may produce results that were originally unforeseen, and these results may ultimately point to practical applications.

Max Perutz explains:

*"Creativity in science cannot be organised. It arises spontaneously from individual talent. Well run laboratories can foster it, but hierarchical organisations, inflexible bureaucratic rules and mountains of futile paperwork can kill it. Discoveries cannot be planned *[8]*."*

Fundamental, curiosity-driven research differs from strategic research in that it is mostly aimed at the fundamental basis of an applied ultimate goal. There will inevitably be arguments over the distinction between fundamental and applied research, as fundamental research in one discipline could easily be taken to mean applied in a different field.

The importance of basic blue skies research is often discussed in scientific circles [9,10], and yet to what extent are funding bodies and the scientific community committed to this process of discovery? Is research in the UK losing out? This study traces the tortuous paths that led to three major medical discoveries, and investigates the extent to which maximising research freedom has influenced scientific discoveries, and the constraints of existing funding on creativity in research, by exploring the views of scientists and medical directors on research direction and freedom.

The term "blue skies research" derives from Julius Comroe, who explained in 1976 how scientific discoveries often arise from tortuous curiosity-driven paths, rather than a direct goal-driven route [11]. He used as his example an event where Charles Wilson, President Eisenhower's Secretary of Defence and an opponent of basic research, said: "I don't care what makes the grass green!" Comroe claimed that Wilson might just as well have said, "I don't care what makes the sky blue!" Comroe defended the need for basic research by describing the work of a British physicist called John Tyndall whose research in 1869 explained the blue colour of the sky by using a glass tube into which he introduced certain vapours. When illuminated, the tube filled with many fine particles. When a powerful beam of light focused on the tube in a dark room, a sky blue cloud filled the tube [12]. Comroe describes how through this discovery, Tyndall's work explained many other unrelated concepts. His examples included the development of a test for optically pure air that was unable to develop bacteria, success in convincing scientists of Pasteur's claim that there was no such thing as spontaneous generation, research demonstrating how lung airways remove particles from inspired air before reaching the alveoli. Tyndall also discovered 50 years before Fleming how *penicillium *bacteria could successfully destroy a mould [13]. He showed how a light beam followed a curved route, leading to the later development of the flexible gastroscope and bronchoscope [14]. Tyndall's work therefore provided strong evidence to show that important discoveries are often curiosity-led rather than goal-driven. Comroe asked US cardiologists to list the professional activities that they considered to be the most valuable. When the origins of these advances were examined, he demonstrated that more than 70% of the clinical tools used by cardiologists arose from fundamental, curiosity-driven research performed without a cardiological outcome in mind. This report was used as a key argument to convince the US Congress that basic blue skies research had a better than average chance of translating into something clinically useful compared to that derived from problem-oriented research [24].

Historically, there has been credibility attached to curiosity-driven knowledge, drawn from its importance held by the ancient Greeks [15]. The value of acquiring knowledge 'for its own sake' continued during and after the scientific revolution. In the early twentieth century, British scientists appealed to the State for support to fund medical research, and this led to the creation of the Medical Research Committee in 1913 allowing some universities to flourish as research centres [16]. In 1918, the Haldane Committee offered scientific freedom, with funding across all university departments [17]. University Grants Committees were formed in 1919, with a system of 'dual support' with two streams of research funding, one stream allocated to teaching, infrastructure and salaries and the other to research. Regular visits by Research Council representatives assessed each university.

In 1971, Lord Rothschild's committee broke away from the traditional view that scientists be allowed freedom from government control, and proposed that research funds be controlled by the government, resulting in a market-oriented approach to research called the 'customer contractor principle' [18]. Since then there has been a gradual shift from scientific freedom towards a focus on objectives in line with the economy or society's expectations [19]. Money from the University Grants Committee became more restrictive and in 1986, the research assessment exercise (RAE) was developed to selectively fund university research [20]. Departments were categorised in terms of their research achievements, leading to UK universities focusing more on developing ways to reflect their performance in RAEs, with a gradual shift away from autonomy. This stifled the pursuit of research excellence by emphasising goals, impacts and outcomes at the potential expense of quality. Dainton believed that this left much to be desired:

*"The system has a distorting effect upon the choice of research topics, causing academics to favour the safe rather than the speculative. In nearly all disciplines, British papers are cited less often than they used to be *[21]*."*

There were those who actively challenged this process. Professor Don Braben, now visiting professor of University College London, worked on nuclear structure and high-energy physics research at the University of Liverpool from 1957 until 1973 after which he held senior positions in the Cabinet Office, the Science Research Council, and the Bank of England. In 1980, he was appointed by British Petroleum (BP) to lead a new research initiative called the Venture Research Unit. Careful selection, minimal paperwork and maximum freedom were key elements of Braben's success in the selection and nurture of several brilliant innovative scientists [22]. Examples of these successes are provided in Appendix 1. This initiative ended ten years later when BP decided to concentrate exclusively on its core business interests. In 1990, Braben set up a new company called Venture Research International Ltd, and continues to campaign for funds to support exceptional innovative researchers who would be allowed scientific freedom, and with it, the opportunity to challenge accepted thinking.

Braben proposed that the successful selection of curiosity-driven research rested on certain elements:

• The research may extend across several national funding committees.

• Objectives should be mostly problem solving.

• The work is innovative.

• The amount of funding may not relate to the likelihood of success.

• The results could radically change accepted thinking.

• The research is not seeking a specific goal, but aims to explore a possibly unique problem.

• The outcome may be uncertain and therefore initial funding may have proved difficult.

• The research is of major interest to all those collaborators involved in the work.

Having the responsibility to explore every curious circumstance is described as pursuing research 'for its own sake' [23]. There are many successful research projects that were not initially seen as having any practical goal, but only much later were applied clinically [24-26]. Success may have derived from the trust and insight of a head of department or funding body, or from a researcher with the 'prepared mind' to apply the findings. Leading scientists may have freedom in the direction of their own research, but younger, less experienced academics may be unable to progress in their career within a peer review system that favours established researchers with a good track record [27].

Some scientists have suggested that talented researchers have been denied the opportunity to explore a topic for its own sake. In 1999, Sir David Weatherall comments:

*"In the few studies that have examined the origins of the major advances in medicine over the past 100 years, it has been clear that a high proportion of them arose from research projects that had no practical goal in sight but which were driven by a simple wish to understand normal physiology. The growing tendency for a more goal-orientated approach to the process of scientific discovery has disadvantages – again it drives scientists towards work that has predictable results *[28]*."*

There are concerns that increased central funding and peer review have obstructed the discovery process. Rather than being curiosity-driven, the research agenda is being managed differently with some research seen as more 'worthy' than others [25]. The Government has tried to retain excellence in UK research with a strategy called 'The Science and Innovation Investment Framework 2004–2014', setting a long-term agenda for UK science and innovation to stimulate collaboration between universities, businesses and industry [29]. The UK Clinical Research Collaboration (UKCRC) in 2004 set up a project to code research activity and classify research into broad categories with rigorous quality control, and to collaborate on issues that cannot be tackled by a single organisation. These strategies, however, appear remarkably similar to the 1993 White Paper 'Realising our Potential', which aimed to enhance investment in basic science research and bring in investments from foundations and corporations. The recent National Health research strategy 'Best Research for Best Health' has a range of objectives, one of which includes support for 'Invention for Innovation programme' to encourage new ideas including a Challenge Fund for Innovation [30]. Again this strategy appears similar to the 2002 Government paper 'Investing in Innovation' that proposed a dedicated capital steam for university science research infrastructure and full costs of the research. And yet concerns about the constraints on scientific freedom persist [31].

This paper focuses specifically on the research climate in the UK, as the restrictions imposed on research in the US and Japan may not be so profound. The US government has offered federal and private sector funding for curiosity-driven fundamental research. However, this funding is renewed annually, reflecting some discomfort about the long-term nature of such research. Congress increasingly requires information from scientists on the direction of their research, the scientific goals to be achieved and whether the advantages to come from this research are socially equal. In the US, the DARPA (Defence Advanced Research Projects Agency) offers funding for radical ideas, and yet they demand that researchers meet clear milestones before funding continues. Recently, the National Institute for Health introduced the EUREKA (Exceptional, Unconventional Research Enabling Knowledge Acceleration) programme – a new funding initiative to help researchers with original ideas, and CEBRA (Cutting-Edge Basic Research Award) to encourage highly innovative research. Although prominent scientists regularly provide political leaders with strong arguments to support curiosity driven research, the lobbying needs to continue annually to maintain the funding momentum. The 2008 budget supporting research in the US has declined [32].

The Japanese government, despite severe economic concerns, has not reduced its expenditure on fundamental, curiosity-driven research, but has actually increased its research expenditure, viewing scientific development as essential for its economic growth [33].

### Examples of the paths taken to achieve major discoveries

When we search for the roots of major scientific advances made over the centuries, they often reveal tortuous routes where the findings bear little resemblance to the initial research. The convoluted paths leading to discovery can be seen in echocardiography, cardiopulmonary bypass, and the discovery of adrenaline, which are described as key contributions to cardiology [34]. These are briefly described below:

**Echocardiography **is one of the most valuable methods of cardiac investigation today. In 1830, Galton's whistle produced sounds beyond the higher limit of hearing, but it was not until instruments to detect ultrasound were developed that the wider value of such a pitch was recognised. In 1913, a physicist called Richardson carried out experiments in testing ultrasound on underwater echo-ranging schemes [35]. In 1917, Langévin, a French physicist, observed that ultrasonic energy could have a detrimental effect upon biological material [36]. Langévin collaborated with a scientist called Wood who was working on ultrasonic waves to detect submarines. During the War, Britain and France asked Wood to join other leading scientists, because of his skills at technical innovation whilst working on telescopes. There, Wood met a scientist called Loomis. They both saw the potential in pursuing Langévin's work, and their subsequent research revealed that exposure to ultrasonic energy had a powerful impact on displacing material. Two brothers, Karl Dussik a neurologist, and Freiderich, a physicist, studied the clinical application of ultrasound in 1937, by measuring an ultrasound beam through the skull. They used this technique to look inside the human body, and in 1942 Dussik used echocardiography to measure phasic volume changes of the heart [37]. Therefore, the development of echocardiography came from a winding and curiosity-driven process. The input came from physicists and engineers whose focus was on solving a problem with an unforeseen path, but only ultimately led to the clinical application of ultrasound.

The development of **cardiopulmonary bypass **can be attributed to the work of Verney and Eichholtz who, from 1921 to 1924, studied the mechanism of urinary secretion [38]. They needed to isolate the kidney to explore the vasoconstriction that occurred whenever they perfused the kidney, and used a heart lung pump developed by Starling to achieve controlled blood flow and temperature for their animal experiments [39]. De Burgh Daly modified this pump, which later formed the basis of Clarence Dennis's research into cardiopulmonary bypass for patients undergoing heart surgery [40]. Dennis met John Gibbon, who had been working in this area for several years and they subsequently collaborated to eventually perform the first successful heart lung by pass procedures [41]. The research leading to this final application was mainly exploratory and curiosity-driven. The outcome could not have been envisaged at the outset, and would have been unlikely to achieve with a goal driven proposal.

The discovery of the effect of adrenaline on blood pressure by Edward Schäfer (1850–1935) has its roots in the work of Oliver (1841–1915), a physician from Harrogate, who often spent his leisure time carrying out experiments on his family. Oliver was interested in developing an instrument to measure the diameter of the radial artery. He had an idea that patients with low blood pressure might benefit from extracts of the adrenals. He therefore gave his son an injection of extract from the adrenal medulla prepared from material supplied by the local butcher, and thought that he saw the radial artery contract. He travelled from Harrogate to University College London to describe his discovery to the physiologist Professor Schäfer, who was not impressed with Oliver's observation but agreed to use the extract in his experiment on a dog. To his amazement Schafer saw the mercury climb dramatically in the blood pressure manometer. Their subsequent collaboration led to their demonstration of the effect of adrenaline on blood pressure in 1894 [42]. It was Oliver's curiosity, and Schäfer's ability to collaborate with a physician and change the direction of his research, that allowed this discovery to be made.

These three discoveries are unlikely to have been made if they had demanded goal-driven research proposals with tight time scales, and yet they could have met the criteria used by BP's Venture Research Unit. The objectives were mainly problem-solving, the funding support bore little relation to the likelihood of success, the results radically changed accepted thinking; there was no clear time-frame, but a need to understand a problem. Many other key developments have followed similar tortuous paths, such as the role of aspirin, the structure of haemoglobin, the LASER, molecular biology, and coronary angiography, all of which led to outcomes that were not envisaged at the outset.

## The Interviews

### Methods

This study gathered the views of eight renowned scientists and medical directors on past and existing research direction and on the extent of freedom needed to allow science to flourish. The interviews involved questions about their views on the selection, funding, quality and process of discovery, on research freedom and constraints on scientific progress in the UK. A qualitative grounded theory approach was used to create categories and concepts to explain trends in thought and offer a guide to action [43]. The interviews were based on questions arising from the literature review, which are outlined in Table 1. However, an informal unstructured interview was preferred to a quantitative survey as the aim was to explore the views of scientists and medical directors by allowing free discussion of issues that may have been unforeseen. Each interview lasted no longer than 30 minutes.

**Table 1:** Questions to interviewees drawn from the literature reviewed

Selection	Pressures on researchers to anticipate their goals in research applications	Whether a research application was necessary before 1970	The impact of the peer review process on developments in research	Whether research before 1970 would be accepted with the current peer review system
Funding	Priorities for funding	Changes in the funding environment within the UK	Ways to manage limited funding sources in the UK	
Quality of research	Experience of cross disciplinary working	Interpretation of the term blue skies research	Financial support to be allocated to blue skies research	
Process of discovery	The commercialisation of research activity	The ingredients in a researcher and the environment needed for discovery	Personal experience of the environment that influenced the process of discovery	

The interviewees were selected because they were all influential leaders in their area of work. They had a great interest in research, were involved in making funding decisions and were responsible for making the best use of research money, and included scientific advisors, Heads of Departments, Fellows of the Royal Society, Fellows of the Royal College of Physicians, and a Nobel Laureate. Two were former medical directors who had more clinically orientated backgrounds. Two were medical directors with more scientific leanings. Four scientists were involved in cardiovascular research, pharmacology, pathology or respiratory physiology. The backgrounds of these key specialists are described in Table 2.

**Table 2:** Profile of Interviewees

**Interviewee**	**Location**	**Speciality**	**Department affiliation**	**Gender**	**Orientation: scientist or clinician**
1.	Cambridge	Cardiovascular	University professor and Medical Director	Male	Scientist and clinician
2	Birmingham	Cardiovascular	University professor Medical director	Male	Clinical
3	Southampton	Pharmacology	University professor Medical Director	Male	Clinician
4.	Oxford	Cardiovascular	University professor	Male	Scientist
5	London	Cardiovascular	University professor Medical Director	Male	Scientist and clinician
6	London	Oral Pathology	University Professor	Male	Scientist and clinician
7	London	Respiratory Physiology	University Professor	Male	Scientist
8	London	Pharmacology	University professor Nobel Laureate	Male	Scientist

One-to-one interviews were arranged, after letters explaining the project were sent to the interviewees, who agreed to have their interviews recorded. One interviewee discussed his views over lunch, while another gave his views in writing. The information from the transcripts was classified into tables, and then coded using colour and letter coding to distinguish recurring themes that emerged from the interviews. Table 3 outlines the layout used to organise and colour code the themes emerging from the interviews.

**Table 3:** Tables used to tabulate and code the interviewees' comments (completed tables available on request):

Interviewee:	1	2	3	4	5	6	7	8
Academic freedom								
Academic freedom & the future								
Demands for goal-oriented research?								
Selecting the innovative thinker								
Qualities of blue skies researcher								
Funding								
Proportion of funding as curiosity driven								
Providing opportunities								
Short term vs. long term								
Funding across disciplines								
Commerciality								
Peer review								
Peer review – restrictions								
Examples of freedom						.		
Examples of discoveries in research								
Changes since 70s								
Expectations of the public								
Expectations of the organisations								
Terminology								,

## Results

The data obtained from these interviews built a picture of the issue of basic, blue skies research from the interviewees' perspective. The interviewees gave their views on broad topics such as: academic freedom, funding, personal experiences, the constraints of goal-orientated research, selection and management of innovative research, and external influences affecting the research process. These themes are discussed in turn, explaining the majority views of the interviewees.

### Academic freedom

The interviews reflected an overall consensus over the meaning of blue skies research as curiosity-driven, innovative, uncertain of where the research was leading, and with an unclear time frame. Individual curiosity was seen as something within the researcher, as described by this scientist:

" Essentially curiosity is something that resides within an individual. The most successful laboratory in biology in the world judged by Nobel prizes is the laboratory of molecular biology in Cambridge. The man who ran it is on record as saying that all they did was interview you and if they liked what you were talking about, they would say: 'Right, here's your money. All you have to do is to come into tea in the afternoons and tell us what you're getting on with, and talk about it' and you just got ahead."

A pathologist also illustrated the importance of academic freedom:

"Something to be revered and fought to maintain, against ever-increasing mechanistic pressures to conform to job descriptions and other restricting influences."

### Funding of innovative research

The interviewees believed that while they saw a place for goal-orientated research, they also felt that some funding should be channelled towards curiosity-driven research, which could hold the key to future discoveries, as clarified by one scientist:

"I'm not asking that this is the only way that people should be funded. But I'm simply asking that there should be a proportion. That amount of funding could be set aside and administered differently. It is based upon trust, and the trust is based upon the ability of the researcher to infect you with their enthusiasm."

Most scientists felt that five years of research funding would provide enough evidence to reveal how well researchers are spending the grant, and whether the research warranted further support. Short-term contracts may not offer the security needed to encourage researchers to invest their time in curiosity driven research. One scientifically orientated medical director described the potential impact of short term funding on innovative research:

"Without any doubt, the funding that has been most effective from my point of view, has been that which goes on for at least five years. If you've got to justify yourself after one year, you won't be doing anything more than next step research."

Many innovative discoveries cut across various disciplines, even though most UK funding sources are restricted to one speciality. The interviewees felt that there should be more cross speciality funding for innovative research. A medical director describes the issues:

"The scientists don't care and neither should the funders – unless the research drifts too far away from your specific remit as a funder. And that's where the single speciality funders like us are in trouble."

### Personal experiences

The scientists' experiences revealed how constraints imposed by research proposals, such as outlining a possible goal and time frame, and focusing on a specialist interest, denied researchers the opportunity to explore a problem without a particular application in view. They gave examples of applying for 'safe' funding, rather than pursuing what they really felt to be important, and of 'game-playing' by applying for fundable projects, but using the money for more innovative research. Some people may not consider this to be a matter for concern, but the public who often donate the funds to support research need to appreciate the contribution that basic blue skies research makes towards major research discoveries. This reinforces the importance of a clear explanation to the public and policy makers about curiosity-driven research. These views concur with the literature describing the impact of the research assessment exercise, tight time frames on creativity in research [44].

The scientists' personal examples of research findings that bore little resemblance to the initial plan reflected how their research path could not have been anticipated and would have been difficult to submit as a proposal.

One scientist describes his experiences:

"We started by wanting to understand the role of smooth muscle cells in atherosclerosis. We used a technique of genetic hybridisation and found that smooth muscle cells behaved very much like bone cells under certain circumstances. This wasn't what we were interested in to begin with but we pursued it. And out of that initial experiment in 1999 we identified a whole bunch of genes. It took about 10 years to work out what one of the genes was and that's led to a whole new area of biology which has shown that that protein converging may be brought to conditions where patients die prematurely of cardiovascular disease."

They also gave examples of the freedom needed to involve researchers from unrelated disciplines and described how difficulties could occur when a research proposal is tailored to fit a specialist interest:

"As an oral pathologist, I have done research with surgical colleagues involved in anal and rectal tissues, aiming to help increase understanding about Hirschprungs disease. This caused raised eyebrows in the dental school that employed me."

### The constraints of goal-driven research

The discussion of goal-driven research focused on its constraints, which could lead researchers to apply for safe research, and on the process of game playing that occurred when spending the grant. It became clear from the interviews that constraints were imposed on the innovative researcher by goal-driven research applications. Therefore, the funding may need to focus on one avenue of research even though the scientist may see that the research could be of more value if it were allowed to move in another direction. If the goals are clearly outlined and the money sought from one speciality or organisation an innovative researcher may not be able to follow a curiosity-driven path, as the research can only be planned if one knows what the likely outcome is! A clinically orientated medical director explains:

"If you say 'I want to do a project on this particular cell-signalling system to show that (a) goes to (b) goes to (c) and it will cost £100,000', and you give them £100,000, you have a duty to insist that the project is well planned, and properly funded."

The scientists described how promising researchers sometimes coped with the current funding system. They would find it easier to apply for a project more likely to be funded, even though this practice could deny them the opportunity to do what they really wanted to do:

"The system demands this – the researcher knows this. I used to ask PhD students and Post Docs the simple question: 'Are you working on what you really, really want to work on?' And very, very rarely did I get the answer 'yes'. The answer to my question is usually 'I'm working on what I get funded to do. I have kids at school, family etc. And I have to survive – I have to apply for what I can judge to be fundable. And how do I judge that? By looking at what is already funded.' So the system corrupts. The system itself isn't corrupt – but it is corrupting."

One scientist explained how he dealt with the restrictions imposed with goal-driven proposals:

"And so I partly used money that I had managed to get to also do the work for which I knew I couldn't directly get money if I'd submitted a research proposal – this is primarily the reason. And I know from experience, that when I have applied in the past by putting grants in, I was simply told that it was far too ambitious, you couldn't do it! They didn't see where I was going. They didn't know whether I could be trusted, I'm sure, but there you are."

These approaches could perpetuate the problem of inadequate support for curiosity-driven research by disguising the need for freedom for innovative scientists. Despite the accepted need for some progressive research, several scientists felt that the ability of innovative researchers to be creative was neither recognised nor supported:

"I'm not asking for a system where we throw the baby out with the bath water. I'm perfectly happy that there's a lot of progressive work that has to be done, which requires technical skills and knowledge. What I'm worried about is that some individuals are losing out. And we as a consequence as a society are losing out."

### Selection of innovative scientists

Key attributes of those who select innovative researchers were described as trust, judgement, giving a free rein, picking the person rather than the proposal. Suggested attributes of the innovative researcher included enthusiasm, the ability to challenge accepted thinking and to explain their research in simple terms:

" I would need to be able to get you excited about wanting to do something that may be off the wall. And what we're talking about here is not just basic blue skies, it's about irreverence – about not being prepared to accept the story that's being given."

These views are supported in the literature, which describes similar key elements for innovative scientific thought [6].

A clinically orientated medical director suggested that success with funding could spring from having a prior track record that could then draw the trust, and with it more freedom with spending:

"They usually learn their trade in the department and then begin to apply for support in their own right. That is their opportunity to draw attention to their peculiar worth as a novel thinker."

However, this could stifle the innovative researcher as their chosen project may have already been guided into a more popular, 'safe' line of research.

Another clinically orientated medical director felt that there was not a problem with selection by suggesting that:

"The person who has the hunch will always find funds. Actually I don't think fundamentally there are all that many obstacles – you just have to know what the rules are and go along with them."

This approach exposes the difficulty in deciding how much of the current limited resources should be devoted to innovative curiosity driven science, given the current expensive nature of research.

### Managing blue skies research

There was enthusiasm for the continuing value of research, although two scientists contrasted the current scientific climate with research discoveries made before 1970:

"With the dual funding system before 1970, one stream funded the libraries, and all the other things necessary to be the best, and another stream went to the universities, not earmarked, for research, on the grounds that they felt they had a responsibility to make sure that research was going to proceed. Each head of department received his share, which he divvied up among his staff. Some people would do wisely and some would not. You can't tell. Wisdom is a retrospective judgement. You can't warn them, but the people that I'm talking about, such as Huxley and other scientists – they got the money, which came through the department, and they never applied for a grant. Now it's true that research was cheaper in those days – workshops were part of our training, but that isn't really the issue. It's the principle rather than the amount of funding."

If the scientific community supports more curiosity-driven research, it is important to explore how this should best be managed. The scientists and clinicians acknowledged how difficult it is to find people who were unbiased and had the knowledge to assess new innovative research, as described by a scientifically orientated medical director:

"It is actually very, very difficult. There was a time with molecular biology, which was then very new, when there were hardly any people in a position to judge the quality of the work. I remember one particular project about ACE genes, which was turned down, but subsequently proved to be extremely well done. It was initially turned down because the chap who was acting as the chair had his own bias. And this is a very dangerous situation. It is very difficult to get people who are small, but rapidly developing, to benefit from unbiased assessment."

The constraints that the RAE and peer review process impose on the selection of research applications by reviewers, are encapsulated in these comments by two scientists:

"Absolutely disastrous! Peer review has destroyed interdisciplinary research. We don't know where to put that kind of research money."

"Now, supposing that you're one of the reviewers and it's not your money. You wish to be responsible and so you prefer a group application to an individual application because that increases the odds. You prefer something about which a lot can be written as distinct from something that is a bit thin because that spreads the best. You prefer that you're more internationally known and so you choose something that is already recognised internationally. It's not that these people are wicked – the people who sit on these panels are doing the best that they know how. They've been asked to do a job."

Both clinicians and scientists recognised the need for an alternative system to assess, support and manage curiosity driven research projects allowing researchers more freedom than the existing system allows. Most interviewees felt that five years would allow the research to show its potential, after which time further funding support could be provided.

### External influences on scientific freedom

The philosophy of some UK universities such as Cambridge is "the pursuit of education and research at the highest level of excellence, with a core value of freedom of thought and expression [45]." However there were concerns that this philosophy may have been overtaken by commerciality within some UK universities that need to more funding:

"I think the universities have not held their intellectual properties very well. I spent half my life where you sign away your rights on day one. And so there's never a relationship between the money you get and what you do. I may do something that ends up creating a lot of wealth, but I would get no share of that and quite rightly too. So one's judgement is never involved in saying: 'that's more likely to be profitable'. I fear that this might be a problem in universities today. Judgements are now being made about what looks as though it might make money. And that's not what universities should be about."

The interviewees believed that it was important to be honest with the public about justifying their financial support for researchers. They were sometimes under pressure from the media and the public to offer an anticipated practical application and a time frame, which may encourage bold unsupported claims.

"We have to explain it, you know. After all the money has been given by donors, you owe it to them to provide a clear indication as to why the reviewers and the panel members should actually make an award."

Throughout the discussions the scientists were concerned about the development of a culture where researchers may pursue a fundable project, rather than seek research to further understanding.

### Differing views between scientists and clinically orientated medical directors

The scientists were on the whole concerned about the future of curiosity driven research for gifted scientists in the UK. If young scientists are trained in a goal-orientated environment, the potential for them to undertake innovative research may be hampered. The clinicians were less critical of the existing system and felt the allocation of funding to be about right. As those who influence grant provision may be clinically orientated, they may, despite their awareness of the importance of curiosity driven research, opt to focus on research proposals that can be more easily evaluated.

## Discussion

These interviews have exposed blue skies research as holding the key to many discoveries, but suggest that it is still poorly represented. The interviewees recognised the need for a clear and measurable framework with some research, but also acknowledged that inquisitive free-ranging research should be managed in a different way from progressive research.

There was a consensus on the meaning of blue skies research as curiosity-driven, innovative and having a hunch. Interpretations differed slightly, although the terms 'basic' research and 'blue skies' research were often used interchangeably. Curiosity and academic freedom played important roles in innovative research, indicating a continued credibility attached to 'pure' knowledge.

The personal experiences of curiosity driven research described by the scientists revealed that their findings often bore little resemblance to their original purpose. They explained how a grant proposal often needed to be tailored to fit the speciality of the funding source. They described the constraints imposed by research applications, such as the need to outline an anticipated goal and time frame, and to identify a specialist interest, all of which denied gifted scientists the freedom to change direction. They gave examples where scientists would apply for 'safe' funding, rather than pursue an area that they really felt to be important, and where the researcher would submit a proposal that was goal driven and likely to be funded, but used it to pursue more innovative research. This reinforces the need to ensure that the public, the media and policy makers understand the benefits to be gained from basic blue skies research projects.

Key attributes of those who select innovative researchers included trust, judgement, giving a free rein, picking the person rather than the proposal and encouraging younger scientists. Attributes of the innovative researcher included enthusiasm, the ability to challenge and inspire, and to explain their research in simple terms. There was also concern that universities may be seeking profit more than following their original purpose such as the pursuit of excellence.

### Limitations of the study

This was a small study, which sought the views of eminent researchers and fund providers to build a picture of the issues surrounding blue skies research from their perspective. Although they were selected because of their wide experience and areas of influence, most of the interviewees were retired or nearing retirement age. Therefore these views may not be representative of younger scientists.

## Conclusion

Blue skies research forms a vital part of scientific discovery – the interviews provide evidence to show that it is highly regarded by scientists. Despite this, the trend towards research accountability has created constraints, with blue skies research being squeezed out of the equation. The current evaluation process does not allow for freedom, with tight time scales, and proposals requiring an outline of the likely findings. Most research money is speciality-orientated which is not conducive to freedom of direction and course of the research process.

A process of "game playing" may have allowed the research community to mask the constraints affecting innovative research. Scientists may apply for work that is fundable and safe, rather than applying for curiosity based research. Some researchers use grants meant for other research to carry out innovative work -which serves to disguise rather than highlight the problem. While there is a clear need for progressive research, there are also concerns about a lost avenue for blue skies research. Some funding should be made available to attract gifted individuals, who can have the freedom to explain what they would really like to do with the money.

But why is blue skies research not being promoted if it was seen as so vital?

Four possible explanations are:

Firstly, the underlying value behind scientific research 'for its own sake' seems to have lost its way. If this principle were considered important, it would underpin the way in which some funding could be directed of blue skies research. The turning point in the UK was when, in 1971, a market-orientated approach to research was introduced and with it, a shift towards science focusing on objectives in line with economy and more government control.

Secondly, a culture of commerciality among universities has developed since the 1970s with universities experiencing long-term funding problems. This is reflected in mission statements of some universities such as Coventry and Liverpool [46]. It has been suggested that the customer-contractor principle led to a stifling of academic freedom with universities becoming businesses rather than seats of learning or discovery [47]. The introduction of student fees within universities may also have influenced a business culture within universities.

Thirdly, efforts to encourage innovative research cannot use the same assessment processes as for other types of research. Suggestions from interviewees include an ability to inspire the selector and to enthuse over their interest, and where there is complete trust and a freedom to pursue their own interests. These attributes need different measurement tools, and a change in attitude among funders about blue skies research even though the amount of funding needed to do this could be minimal.

Finally, scientists are not always directly involved with distributing grants. The public are vital contributors and may have a desire for researchers to outline their goals. The public, politicians and the media should be made more aware of the importance of blue skies research to reach a discovery, rather than follow a constrained path. Until scientists are willing to explain the vital role of blue skies research on discovery, the public cannot be expected to entrust their donated money to offer such freedom.

We need to develop an innovative approach to assess and manage blue skies research. More interaction between researchers and funding agencies could be encouraged, as government agencies have been keen to prioritise research strategies [48]. Each funding body should have some financial support for blue skies research, but with a specific assessment process that can reassure grant givers of its wider accountability, and which, if unsuccessful, would not be continued. If freedom is so important to science, researchers have a responsibility to help the public and policy makers understand the wider accountability that basic blue skies research holds, despite a lack of short-term gain. A proportion of research funding should be available without short-term expectations, and organisations should promote research in a transparent way, rather than compelling scientists to divert money that was intended for another purpose.

Cardinal John Henry Newman (1801–1890) who created the Catholic University in Dublin propounded a definition of the function of the ideal university as the pursuit of knowledge for its own sake [49]. If the business of universities does not become university business, universities may return to their main purpose – the pursuit of truth and knowledge. We therefore need to explore the values and priorities in universities and society in order to justify basic blue skies research. This could balance the research ethos in centres of learning. Universities could offer lectures on the potential of innovative research to revive enthusiasm for individual curiosity, and with autonomy that would allow some gifted scientists the opportunity to change direction and follow their nose.

## Appendix 1

### Examples of the research arising from the Blue Skies Venture Research Unit [50]

M. Bennett and PH-Harrison were the first to expose the significance of three-dimensional structure within the cell nucleus.

Heslop-Harrison JS, Bennett MD. **Nuclear architecture in plants**. *Trends in Genetics *1990, **6 **: 401–4

P Broda and team pioneered the study of bacterial and fungal genes and enzymes that recycle the main component of plant material called lignocelluloses. They found new ways to trigger gene expression in lignin attack, and discovered enzymes that degrade lignin in grasses and straw, which could improve the nutritional value of traditional animal feeds.

Broda P et al **Lignin biodegradation – a molecular biological approach ***Essays Biochemistry *1985, **24**:82–84.

T Clark and team showed the link between quantum-mechanical behaviour and macroscopic objects, with major implications for future electronic systems, currently based on classical or semi-classical science. The group found new electronic techniques for characterizing weak electronic signals with high spatial resolution, which have many applications in the biological fields.

Widom A, Srivastava Y, Clark TD: **Two-level model of macroscopic quantum tunneling with classical damping ***Phys. Rev*.1993 **B 47**, (22) 15358 – 15359

A Curtis and C Wilkinson studied electrical signal exchange between nerve cell networks and micro-fabricated external electrodes. Leading to the ability to carry out a two-way conversation with small groups (4–5) of nerve cells.

Curtis, A., Wilkinson, C. and Breckenridge, L. **Living nerve nets. **In *Enabling technologies for cultured neural networks*.. Edited by McKenna TM, San Diego: Academic Press, 1994: 99–120

S Davies identified highly efficient ways of synthesizing pure organic-molecules. The work led to the formation of a profitable company called Oxford Asymmetry Ltd.

Davis SJ; **Working paper series**: **Allocative Disturbances and Temporal Asymmetry in Labor Market Fluctuations**,*Economics and econometrics*; Chicago: H.G.B. Alexander Foundation, 1986, 86–38

EW Dijkstra, N van Gasteren, and L Wallen worked to improve on the systematic design of algorithms and mathematical proofs. Such an improvement was needed to make program design more reliable. This 12-year work made a considerable impact on BP's computer-based capabilities.

Dijkstra EW, van Gasteran AJM: **A simple fix-point argument without the restriction to continuity ***Acta Informatica *1986, 23: 1–7

P Edwards and D Logan investigated the electronic phase transitions such as between metals and non-metals, and to explore transitions to a new insulating phase consisting of atoms possessing permanent electric dipole moments, which will enhance our understanding of the behaviour of high-temperature superconductors.

Edwards PP, Johnston EL, Rao CNR, Tunstall DP, Hensel F **The Metal-Insulator Transition: A Perspective ***Philosophical Transactions: Mathematical, Physical and Engineering Sciences*, 1998, **356**:1735.

N Franks and J Deneubourg were the first to quantify the rules over distributed intelligence in ant colonies. As a result of this work, ant colonies are now seen as ideal model systems for understanding information flow and decision-making in biological organizations in general.

Bonabeau E, Theraulaz G, Deneubourg JL, Franks N, Rafelsberger O, Joly JL, Blanco S: **A model for the emergence of pillars, walls and royal chambre in termite nests**, *Philosophical Transactions of the Royal Society of London Series B*, 1998, **353**:1561–1576.

D Herschbach pioneered the use of dimensional scaling as a route to calculating the electronic structure of atoms and molecules. He found that it led to remarkable simplifications in calculating systems that are arduous or intractable using conventional techniques. Recently, the new technique has also proved effective in treating boson correlations in the growing field of Bose-Einstein condensates.

Lopez-Cabrera M, Goodson DZ, Herschbach DR, Morgani JD: **Large-order dimensional perturbation theory for H**_2 _*Phys. Rev. Lett*. 1992, **68**: 1992 – 1995

J Kimble studied the quantum dynamics of optical systems, and, among other things, pioneered new ways of producing squeezed quantum states of light. This has led to an area of research that allows non-linear optics to be performed at the level of single atoms and photons.

McKeever J, Buck JR, Boozer AD, Kimble HJ: **Determination of the Number of Atoms Trapped in an Optical Cavity**, *Phys. Rev. Lett.*2004, **93: **143601

G Parkhouse was a "theoretical engineer" who developed a new integrated approach to the theory of structures showing brittleness to be an advantageous property in structural design. His developments in describing and controlling performance could impact upon the design of new materials and composites.

Parkhouse JG, Kelly A: **The regular packing of fibres in three dimensions.. ***Proceedings of the Royal Society of London Series A-Mathematical Physical and Engineering Sciences *8 Jul 1998 454A: 1889–1909. Q41 RSLP (CL & SC Bound Periodicals)

A Paton, A Glover, and E Allan pioneered the development of novel symbioses between bacteria and plants. The group explored a form of genetic exchange that may have been important in the evolution of life forms. This may open up a wide range of scientific and technological possibilities, such as plants used as bioreactors, and disease resistance.

Amijee F, Allan EJ, Waterhouse RN, Glover LA, Paton AM: **Non-pathogenic association of L-form bacteria (Pseudomonas syringae pv. phaseolicola) with bean plants (Phaseolus vulgaris L.) and its potential for biocontrol of halo blight disease**. *Biocontrol Science and Technology*.1992, **2: **(3) 203–214.

M Poliakoff explored the use of super-critical fluids as an environment for reaction chemistry. His work has led to a greater understanding of these unusual fluids, and to the development of completely new industrial processes. These novel fluids may offer important advantages in the use of bio feedstock as a source of organic chemicals such as plastics.

Darr JA, Poliakoff M: **New Directions in Inorganic and Metal-Organic Coordination Chemistry in Supercritical Fluids**.***Chem Rev*. **1999, **99**(2): 495–542

A Rayner and team and Ian Ross and team initially working independent, these two teams saw common objectives while looking at the potential role of mitochondria in regulating cell behaviour and programmed cell death (apoptosis). They work could help to explain the apparent random nature of causes of death, even among close siblings and clones

Ross I: **Mitochondria, Sex, and Mortality ***Ann. N.Y. Acad. Sci*. 2004, **1019: **581–584

P Rich found a physical chemical understanding of how biological quinones function, and went on to show the mechanisms of several key enzymes involved in biological energy provision.

Rich PR, Madgwick SA, Brown S, vonJagow G, Brandt U: **MOA-stilbene: A new tool for investigation of the reactions of the chloroplast cytochrome *bf *complex ***Photosynthesis Research *1992, **34:(**3) 465–477

K Seddon discovered the potential of performing chemistry in an ionic environment, compared to an aqueous or organic one. He realised that one could design ionic environments to optimise particular chemical reactions, leading to novel "green" processes, and non-polluting alternatives to conventional solvents.

Seddon K: **Ionic Liquids for clean technology – a review, ***Journal of Chemical Technology and Biotechnology *1997, **4**:351–356.

G Stanley and J Teixeira studied the structural and dynamic properties of confined water, which are different from those in the bulk state. The group discovered a second critical point in liquid water, which feature explains the long puzzling anomalies found experimentally

Bosio L, Teixeira J, Stanley HE: **Enhanced Density Fluctuations in Supercooled H**_2_**O, D**_2_**O, and Ethanol-Water Solutions: Evidence from Small-Angle X-Ray Scattering ***Phys. Rev. Lett*. 1981, **46: **597 – 600

C Self et al studied the role of instability in biological systems. They were the first to photo-activate an antibody, a vital step towards antibody-directed therapy in medicine.

Self CH, Thompson S: **Light activatable antibodies: Models for remotely activatable proteins *Nature Medicine***1996, **2**: 817 – 820

H Swinney and P DeKepper and team were the first to produce the chemical spatial patterns predicted in a classical paper by Alan Turing in 1952. They were also the first to study the space-time evolution of patterns – the spatial distribution of reaction products in chemical systems.

DeKepper P, McCormick W. D, Noszticzius Z, Swinney H.L: **Bubble-Free Belousov-Zhabotinskii-Type Reactions**. *J. Phys. Chem*. 1987, **91**: 2181–2184

R Tucker and team pioneered a mathematical reformulation of inherently non-linear phenomena offering new avenues within gravitation and quantum field theory. Their work was relevant to a wide range of industrial problems, such as the stability of bridges under wind and rain excitations, and fatigue damage to undersea structures.

Tucker WR. Wang C: **On the Effective Control of Torsional Vibrations in Drilling Systems ***Journal of Sound and Vibration *1999, **224: **(1) 101–122
